# The Role of Mindfulness Decompression Therapy in Managing Acute Stress Disorder in Traumatic Fracture Patients

**DOI:** 10.62641/aep.v53i1.1668

**Published:** 2025-01-05

**Authors:** Xiujun Chen, Ci Tian, Yan Zhang, Yangmu Fu, Wuxiang Han, Rong Zhang

**Affiliations:** ^1^Surgery Department, The 305th Hospital of the PLA, 100017 Beijing, China; ^2^Urology Department, The 305th Hospital of the PLA, 100017 Beijing, China; ^3^Orthopedics Department, Hainan Branch, PLA General Hospital, 572013 Sanya, Hainan, China; ^4^Orthopedics Department, The 305th Hospital of the PLA, 100017 Beijing, China; ^5^Nursing Department, The 305th Hospital of the PLA, 100017 Beijing, China

**Keywords:** mindfulness-based stress reduction therapy, traumatic fractures, acute stress disorder, psychological state, quality of life

## Abstract

**Background:**

Traumatic fractures are common orthopedic injuries with higher incidence globally, leading to acute stress disorder (ASD). Therefore, this study aimed to analyze the clinical outcomes of mindfulness-based stress reduction (MBSR) therapy in patients with traumatic bone fractures suffering from ASD.

**Methods:**

This study included 135 patients who underwent trauma and fracture treatment at The 305th Hospital of the PLA between August 2021 and August 2023. Based on their participation in MBSR therapy, they were categorized into a conventional group (n = 62) and a combined group (n = 73). We comparatively analyzed the ASD Scale (ASDS), Self-Rating Anxiety Scale (SAS), Self-Rating Depression Scale (SDS), Self-Rating Sleep Status Scale (SRSS), and World Health Organization Quality of Life (WHOQOL) measurement–BREF (WHOQOL–BREF) scores between these two experimental groups. Furthermore, we assessed the incidence of ASD after treatment between these two groups.

**Results:**

There were no significant differences in gender, age, body mass index, education, income, type of expense, trauma type, marital status, fracture site, diabetes status, hypertension status, and the pain visual analog scale (VAS) score, activities of daily living (i.e., modified Barthel index) score, and Social Support Rating Scale score between the two experimental groups (*p* > 0.05). Moreover, no significant differences were found in the prevalence of ASDS before treatment between these two groups (*p* > 0.05). However, after treatment, the ASDS score was significantly lower in the combined group than in the conventional group (*p* < 0.05). Furthermore, post-management analysis revealed that the incidence rate of ASD was 24.19% in the conventional group and 8.22% in the combined group. Moreover, the incidence of ASD was significantly lower in the combined group compared to the conventional group (*p* < 0.05). Before intervention, the difference in the SAS or SDS between patients was not statistically significant (*p* > 0.05). However, following treatment, the SAS and SDS scores of patients were significantly lower in the combined group than in the conventional group (*p* < 0.05). Similarly, after treatment, the SRSS scores of patients were substantially lower in the combined group than in the conventional group (*p* < 0.05). Furthermore, the WHOQOL–BREF score of patients was significantly greater in the combined group than in the conventional group (*p* < 0.05).

**Conclusion:**

MBSR therapy can significantly alleviate ASD in trauma and fracture patients. Furthermore, this approach can alleviate the incidence of ASD and reduce anxiety, depression, and negative emotions in patients. These positive effects collectively improve sleep quality and overall well-being of patients.

## Introduction

Traumatic fractures are among the prevalent types of orthopedic injury worldwide, with high incidence rates. Due to the ongoing advancements in transportation and industry in recent years, the incidence of traumatic fractures has steadily increased. These traumatic fractures not only inflict severe physical and psychological consequences for patients but also have profound repercussions. Consequently, many patients experience varying degrees of acute stress disorder (ASD) [1, 2]. ASD is characterized by an acute stress response occurring within one month of the traumatic event. Symptoms of ASD include traumatic experiences, persistent tension, irritability, and sleep disorders. This condition exacerbates the pain and affects daily activities. In severe cases, ASD can lead to the development of post-traumatic stress disorder (PTSD), significantly affecting the overall prognosis of patients [3, 4]. Current studies have indicated that the prevalence of ASD among patients with traumatic fractures is considerably higher than in the general population, with approximately 28.20% of patients developing ASD [5, 6]. Given this high incidence, managing the mental health of trauma and fracture patients is crucial. Close monitoring of their mental state and providing effective nursing interventions can improve the acute stress state and reduce the occurrence of ASD, ultimately enhancing the quality of life.

Mindfulness-based stress reduction (MBSR) therapy, also known as mindfulness meditation, is a systematic meditation training method derived from traditional Buddhism that can reduce stress and enhance emotional management. MBSR therapy focuses on reducing emotional stress and improving psychological resilience by cultivating a present-movement awareness, practicing nonjudgmental observation of one’s experience, and accepting them without resistance [7, 8]. Moreover, MBSR therapy involves mindful breathing, mindful yoga, and mindful walking.

Concentration and self-regulation techniques are used to alleviate psychological pressure and improve patients’ ability to manage negative emotions [9, 10]. Positive pressure decompression therapy has been found effective in improving psychological abnormalities after surgery for lumbar degenerative diseases [11]. While existing research supports the effectiveness of MBSR therapy in orthopedic surgery-related populations, its application in patients with traumatic fractures and ASD remains uninvestigated and requires further exploration. This retrospective cohort study aimed to assess the clinical effects of MBSR therapy in trauma and fracture patients. The outcomes of this research can serve as a valuable reference for future clinical practice and further study. By comprehensively understanding the psychological rehabilitation of patients with trauma and fractures, we aim to develop personalized and effective treatments to enhance their mental well-being.

## Materials and Methods

### Basic Information

The study included traumatic fracture patients from The 305th Hospital of the PLA between August 2021 and August 2023. Based on patient’s records and whether they received MBSR therapy, the 135 participants were categorized into two groups: the conventional group (n = 62) and the combined group (n = 73). This study was approved by the Ethics Committee of the The 305th Hospital of the PLA (approval No. KYLL-SPJ-2024-05) and conducted in compliance with the ethical standards of the 1964 Helsinki Declaration and its subsequent amendments. All participants provided informed consent.

### Inclusion and Exclusion Criteria

The inclusion criteria for patients were as follows: (1) meeting the diagnostic criteria for emergency trauma fractures confirmed via computed tomography (CT) or magnetic resonance imaging (MRI); (2) possessing clear autonomous consciousness and good cognitive ability; (3) patients having complete clinical data; (4) being >18 years old; and (5) hospitalized at The 305th Hospital of the PLA for more than four weeks.

Furthermore, the exclusion criteria for patients were as follows: (1) impaired consciousness, intellectual disability, or neurological disease; (2) alcoholism or drug dependence; (3) abnormal function of vital organs such as the liver, lung, or kidney; (4) cerebrovascular disease; and (5) malignant tumors.

### Methods

Both groups of patients received routine symptomatic treatment upon admission. This treatment included wound cleaning, disinfection, anti-infection treatment, rehydration, correction of electrolyte imbalances, nutritional support, and preoperative preparation. Patients in the conventional group received routine care, including condition monitoring, environmental care, trauma care, medication guidance, psychological intervention, prevention of complications and preoperative preparation.

In contrast to the conventional group, the combined group received MBSR therapy care over a four-week nursing process. During the first week, the theory of MBSR therapy and the individualized nursing process were thoroughly explained to the patient in detail. The nursing process is based on the patient’s condition and physical fitness. Furthermore, health education and training manuals were distributed among the patients. The importance of MBSR therapy was emphasized via slide presentations and video playback. Additionally, the patients were encouraged to train for more than 60 minutes daily to face trauma and fractures, building confidence and strategies to overcome their disease condition. Moreover, attention was given to their psychological changes, with timely communication to understand and alleviate their anxiety and depression, helping them realize the normality of adverse emotions. Lastly, patients were guided to maintain an objective attitude and to focus their attention on the feeling of mindful breathing.

In the second week, the method of body introspection was explained. By scanning their own state, patients could establish a connection between their physiological and psychological conditions, developing appropriate associations. The patients were guided to practice mindful breathing and a three-minute breathing space to observe subtle changes in their bodies during breathing. Mindfulness training involving the five senses—sight, touch, taste, hearing, and smell—was conducted once daily for 30 minutes to help patients feel the beauty around them. Furthermore, patients were guided to add 10 minutes of mindfulness meditation training to their routine.

In the third week, the patients were guided to observe and perceive changes in their body and surroundings via meditation and contemplation. Furthermore, they were encouraged to relax the body and mind, engaging in a state of contemplation once daily for 30 minutes to cope with the pressure brought about by their disease condition. This engagement aims to reduce negative emotions such as anxiety and depression, effectively improving their mental state and self-care abilities. Furthermore, they participated in a 10-minute daily mindfulness walking training. In the last week, patients were guided to integrate MBSR therapy into their daily lives and routine activities. Additionally, various techniques such as body scanning and mindfulness observation were utilized to improve their ability to adapt to their environment and enhance their quality of life. Simultaneously, patients were engaged in 40–50 minutes of daily mindfulness stress relief training.

### Evaluation Criteria

Study participants were evaluated as follows:

(1) ASD status: The ASD Scale (ASDS) was used to assess the ASD status of patients [12], with a Cronbach’s R coefficient of 0.960 for reliability and validity. Scores were obtained before and four weeks after management. The quantity scale includes four dimensions: avoidance symptoms, dissociation symptoms, re-experiencing symptoms, and hypervigilance symptoms, totaling 19 items. Each item is rated on a scale of 1 to 5, with a possible score ranging from 19 to 95 points. A higher score indicated the severity of the ASD status. Additionally, we utilized the Diagnostic and Statistical Manual of Mental Disorders, Fifth Edition, as the standard reference to compare ASD incidence rates between the two groups [13].

(2) Mental state: The Self-Rating Anxiety Scale (SAS) was used to evaluate the anxiety level of patients, with a Cronbach’s coefficient of 0.931 for reliability and validity [14]. The Self-Rating Depression Scale (SDS) was used to assess the degree of depression, with a Cronbach’s coefficient of 0.863 [15]. These two scales were administered before and four weeks after management, each comprising 20 items rated on a scale of 1 to 4, with a total score ranging from 20 to 80 points. Higher SAS and SDS scores indicate more severe anxiety and depression symptoms in the patient.

(3) Sleep quality: The Self-Rating Sleep Status Scale (SRSS) was used to assess the sleep quality of patients after management [16], with a Cronbach’s R coefficient of 0.6418 for reliability and validity. The scale consists of 10 questions, each scored on a scale of 1 to 5. The total score ranges from 10 to 50. A higher score on the SRSS indicates poorer sleep quality.

(4) Quality of life: The World Health Organization Quality of Life (WHOQOL) Measurement–BREF (WHOQOL–BREF) was utilized to assess the quality of life of patients following the intervention [17]. The Cronbach’s R coefficient for reliability and validity was 0.880. The scale includes five dimensions: physiological domain, psychological domain, social relationship domain, environmental domain, and overall quality of life domain. A total of 26 items, each rated on a scale of 1 to 5, were considered. The total score ranged from 26 to 130 points. A higher WHOQOL–BREF score indicates a better quality of life.

### Statistical Methods

Data analysis was conducted using SPSS 19.0 (IBM Corporation, Armonk, NY, USA), while Microsoft PowerPoint 2016 (Microsoft, Seattle, WA, USA) was utilized to generate figures. Categorical data such as gender, education, and satisfaction were presented as [n (%)] and analyzed using the chi-square test. Continuous variables such as age, ASDS, and SAS were expressed as mean ± standard deviation ($\bar{x}$ ± *s*). Furthermore, pairwise comparisons were performed using the *T*-test.

## Results

### General Information

There were no statistically significant differences in body mass index (BMI), education level, monthly income, cost type, trauma type, marital status, fracture site, diabetes status, hypertension status, and visual analog scale (VAS) score between the patients in the combined group and those in the conventional group. Furthermore, the modified barthel index (MBI) and Social Support Rating Scale scores did not show significant differences between the two groups (Table 1).

**Table 1.** **Baseline characteristics of the study participants [n (%), ($\bar{x}$ ± *s*)]**.

Indicators		Conventional group (n = 62)	Combined group (n = 73)	*t/χ^2^*	*p-*value
Gender	Male	31 (50.00)	42 (57.53)	0.766	0.381
	Female	31 (50.00)	31 (42.47)		
Age (years)		45.23 ± 6.27	44.88 ± 5.88	0.333	0.739
BMI (kg/m^2^)		22.16 ± 2.08	22.41 ± 2.31	0.657	0.512
Educational level	Undergraduate or above	18 (29.03)	23 (31.51)	0.157	0.984
	Specialist	16 (25.81)	18 (24.66)		
	High school	14 (22.58)	15 (20.55)		
	Junior high school and below	14 (22.58)	17 (23.29)		
Monthly income	<3000 CNY	23 (37.10)	26 (35.62)	3.175	0.365
	3000–6000 CNY	26 (41.94)	23 (31.51)		
	6001–10,000 CNY	9 (14.52)	19 (26.03)		
	>10,000 CNY	4 (6.45)	5 (6.85)		
Cost type	Self-funded	23 (37.10)	28 (38.36)	0.346	0.841
	Medical insurance	18 (29.03)	18 (24.66)		
	Work injury insurance	21 (33.87)	27 (36.99)		
Trauma type	Traffic accident	38 (61.29)	40 (54.79)	0.836	0.659
	Accidental violent injury	10 (16.13)	16 (21.92)		
	Sports injuries	14 (22.58)	17 (23.29)		
Marital status	Unmarried	41 (66.13)	49 (67.12)	1.827	0.401
	Married	15 (24.19)	21 (28.77)		
	Widowed/divorced	6 (9.68)	3 (4.11)		
Fracture site	Femoral fracture	32 (51.61)	36 (49.32)	1.407	0.704
	Tibiofibular fracture	22 (35.48)	25 (34.25)		
	Patellar fracture	5 (8.06)	10 (13.70)		
	Lumbar vertebral fracture	3 (4.84)	2 (2.74)		
Hypertension	Yes	13 (20.97)	11 (15.07)	0.798	0.372
	No	49 (79.03)	62 (84.92)		
Diabetes	Yes	9 (14.52)	6 (8.22)	1.346	0.246
	No	53 (85.48)	67 (91.78)		
SSRS score	Low level	10 (16.13)	11 (15.07)	0.516	0.772
	Moderate level	43 (69.35)	48 (65.75)		
	High level	9 (14.52)	14 (19.18)		
VAS score		6.43 ± 1.21	6.58 ± 1.33	0.681	0.497
MBI score		20.56 ± 6.13	20.67 ± 5.18	0.113	0.910

Note: Measurement data meeting the criteria of normal distribution were analyzed using a *t*-test; Categorical data were analyzed using the chi-square test (χ^2^); BMI, body mass index; SRSS, Self-Rating Sleep Status Scale; VAS, visual analogue scale; MBI, modified barthel index; 1 USD = 6.48 CNY.

### ASD Status

**Fig. 1.**
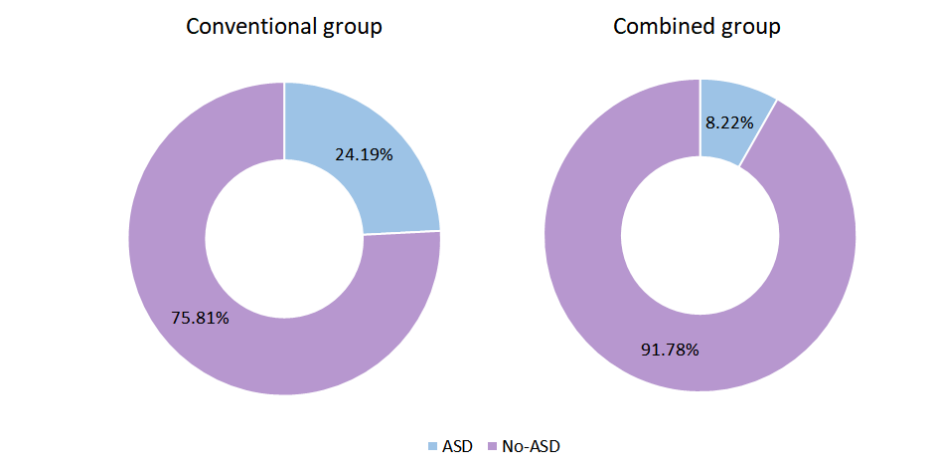
**Comparative acute stress disorder (ASD) incidence between the conventional and combined groups**.

The ASDS scores of patients between the two groups were statistically insignificant before treatment (*p* > 0.05). However, after management, the ASDS scores of patients in the combined group were significantly lower than those in the conventional group (*p* < 0.05). Furthermore, post-management analysis revealed that the incidence rates of ASD among patients in the two groups were 24.19% and 8.22%, respectively. Moreover, we observed that the incidence of ASD was significantly lower in the combined group compared to the conventional group (*p* < 0.05, Table 2A,2B and Fig. 1).

**Table 2A.** **Comparison of the ASDS scores between the two groups ($\bar{x}$ ± *s*)**.

Experimental groups	n	Separation symptoms		Re-experience symptoms		Avoiding symptoms	
		Before	After	Before	After	Before	After
Conventional group	62	9.19 ± 2.33	8.13 ± 2.03	9.03 ± 1.69	8.16 ± 1.07	8.74 ± 2.17	7.66 ± 1.68
Combined group	73	9.22 ± 2.38	7.23 ± 2.07	9.00 ± 1.55	7.22 ± 1.25	8.88 ± 2.07	6.56 ± 1.37
*t*		0.074	2.533	0.108	4.648	0.383	4.190
*p*		0.941	0.012	0.915	<0.001	0.702	<0.001

Note: Measurement data conforming to normal distribution were analyzed using a *t*-test. ASDS, acute stress disorder (ASD) Scale.

**Table 2B.** **Comparison of the ASDS scores between the two groups ($\bar{x}$ ± *s*)**.

Experimental groups	n	High alertness symptoms		Total score	
		Before	After	Before	After
Conventional group	62	15.21 ± 2.12	13.63 ± 2.07	42.17 ± 4.30	37.58 ± 3.17
Combined group	73	15.14 ± 2.88	12.44 ± 2.06	42.23 ± 4.39	33.45 ± 3.53
*t*		0.158	3.337	0.080	7.097
*p*		0.874	0.001	0.937	<0.001

Note: Measurement data conforming to normal distribution were analyzed using a *t*-test.

### Psychological Indicators

The SAS and SDS scores of patients in the two groups did not show significant differences before treatment (*p* > 0.05). However, after intervention, the scores in the combined group were significantly lower than those in the conventional group (*p* < 0.05, Tables 3,4).

**Table 3.** **Comparison of SAS scores between the two groups ($\bar{x}$ ± *s*)**.

Experimental groups	n	SAS score	
		Before	After
Conventional group	62	49.21 ± 7.16	46.89 ± 5.29
Combined group	73	48.79 ± 6.36	41.29 ± 5.11
*t*		0.360	6.243
*p*		0.719	<0.001

Note: Measurement data meeting the criteria of normal distribution were analyzed utilizing a *t*-test; SAS, Self-Rating Anxiety Scale.

**Table 4.** **SDS scores of the two groups ($\bar{x}$ ± *s*)**.

Experimental groups	n	SDS score	
		Before	After
Conventional group	62	52.50 ± 6.26	49.82 ± 5.62
Combined group	73	51.96 ± 5.28	42.14 ± 6.03
*t*		0.545	7.607
*p*		0.587	<0.001

Note: Measurement data conforming to normal distribution were analyzed using a *t*-test; SDS, Self-Rating Depression Scale.

### Sleep Quality

After treatment, the SRSS scores of patients in the combined group were significantly lower than those in the conventional group (*p* < 0.05, Table 5).

**Table 5.** **Comparison of the SRSS scores between the two groups of patients ($\bar{x}$ ± *s*)**.

Experimental groups	n	SRSS score
Conventional group	62	25.74 ± 5.12
Combined group	73	23.97 ± 5.14
*t*		1.997
*p*		0.048

Note: Measurement data conforming to normal distribution were analyzed using a *t*-test; SRSS, Self-Rating Sleep Status Scale.

### Quality of Life

After treatment, the WHOQOL–BREF score of patients in the combined group was significantly greater than that in the conventional group (*p* < 0.05, Table 6).

**Table 6.** **Comparison of the WHOQOL–BREF score between the two groups of patients ($\bar{x}$ ± *s*)**.

Experimental groups	n	WHOQOL–BREF score
Conventional group	62	86.19 ± 6.67
Combined group	73	90.84 ± 5.08
*t*		4.592
*p*		<0.001

Note: Measurement data conforming to normal distribution were analyzed employing a *t*-test; WHOQOL-BREF, World Health Organization Quality of Life Measurement.

## Discussion

Traumatic fractures are characterized by their sudden and unpredictable nature. These fractures not only inflict physical and cognitive damage but also have psychological impacts, leading to significant stress responses. Some patients experience symptoms of ASD when faced with setbacks and adversity [18, 19]. ASD, a stress reaction occurring following severe physical trauma, is characterized by consciousness, cognition, memory orientation, psychomotor behavior, and sleep disorders. Patients may experience insomnia, loss of appetite, daydreaming, irritability, depression, and increased self-consciousness, usually accompanied by abnormal behaviors, which may potentially progress to PTSD [20, 21]. A study has shown that approximately 40% to 80% of ASD patients develop PTSD within six months following a traumatic event [22]. Therefore, addressing post-traumatic ASD symptoms is crucial for improving patient prognosis.

MBSR therapy is an intensive training method based on mindfulness meditation and is used to address chronic pain and stress. This four-week course emphasizes a continuous awareness of an individual’s own state. MBSR therapy has been found to enhance mindfulness, reduce stress, and alleviate anxiety in patients with various medical and psychological problems [23, 24].

In this retrospective clinical study, we aimed to analyze whether MBSR therapy can improve ASD and other psychological and quality-of-life outcomes in patients with traumatic fractures. The differences in ASDS scores of patients between the two groups before treatment were not significant (*p* > 0.05). However, after treatment, the ASDS scores of patients in the combined group were significantly lower than those in the conventional group (*p* < 0.05). The incidence rates of ASD in the conventional and combined groups were 24.19% and 8.22%, respectively. The incidence of ASD in the combined group was significantly lower than in the conventional group (*p* < 0.05). Hence, MBSR therapy, when combined with conventional treatment, can substantially reduce the occurrence of acute stress issues in patients with trauma and fractures. Omidi *et al*. [25] explored the efficacy of MBSR therapy as an intervention for PTSD and reported it to be an effective method for improving the emotional state of veterans who have PTSD. Compared to the study by Omidi *et al*. [25], our study focused on patients’ ASD conditions before PTSD onset and showed the great effect of MBSR therapy, suggesting the importance of patients receiving mindfulness therapy in a timely manner after trauma. MBSR therapy can regulate the sympathetic-parasympathetic nervous system, increase dopamine levels, reduce sympathetic nerve activity, and reduce stress responses through several mechanisms, thereby alleviating the incidence of ASD [26, 27]. Furthermore, the improvement of ASD in patients with MBSR may be related to enhanced activity in prefrontal areas, such as the mPFC, and reduced activity in limbic areas, such as the amygdala. This targeted modulation of neural activity can positively impact intrusion and hyperarousal symptoms commonly experienced by individuals with ASD [28, 29].

The SAS and SDS are common clinical scales for evaluating anxiety and depression, known for their excellent reliability and validity. Standardized scoring standards can better compare individual differences and assess the severity of psychological states, providing a foundation for early clinical evaluation, subsequent intervention, and treatment. In our study, the differences in SAS and SDS scores between the two groups before treatment were not statistically significant (*p* > 0.05). However, after the intervention, the SAS and SDS scores significantly decreased between the two groups (*p* < 0.05). Thus, MBSR therapy can effectively reduce negative emotions, including anxiety and depression, in patients with trauma and fractures. The results of this study align with the findings of Hoge *et al*. [30], who suggested that MBSR therapy is not effective in treating depression. Currently, escitalopram MBSR reduces the reactivity of the autonomic nervous system and enhances the attention mechanism of patients, which can improve their emotional regulation and decrease their personalized reactions to thoughts and feelings. Consequently, individuals can adjust problematic habitual thinking modes, reducing poor emotions such as anxiety and depression. In this study, after undergoing combined management, patients in the intervention group showed a significant increase in their WHOQOL–BREF scores compared to the control group (*p* < 0.05). Reich RR *et al*. [31] investigated the effectiveness of MBSR interventions for breast cancer patients experiencing multiple symptoms. This study aimed to assess the impact of this intervention on the quality of life of patients. The results revealed that MBSR therapy had a significant positive effect on improving the overall quality of life in this particular group of breast cancer patients. MBSR therapy effectively reduced psychological symptoms such as depression, anxiety, stress, and fear of recurrence; physical symptoms such as fatigue, pain, sleep, and lethargy; and cognitive symptoms, contributing to enhanced well-being and quality of life for the patients.

Pain resulting from trauma and fractures can seriously affect not only the sleep quality of patients but also their psychological well-being, such as concerns about the efficacy of surgery, financial pressure, and feelings of guilt related to their condition. Long-term sleep disorders can accelerate metabolism and aggravate psychological stress and physical stress. Patient discomfort significantly affects various aspects of surgical treatments and overall quality of life, potentially impacting patient prognosis [32].

This study revealed that following intervention, the SRSS scores of patients in the combined treatment group were significantly lower than those in the conventional treatment group (*p* < 0.05). These findings suggest that MBSR therapy can be beneficial for improving sleep disorders in trauma and fracture patients. The findings of this study are consistent with those of Liu *et al*. [33], who investigated a group of 112 patients with osteosarcoma to determine the effects of MBSR therapy on sleep quality. The findings revealed a positive impact of MBSR therapy on improving patients’ sleep quality. Diez *et al*. [34]demonstrated the potential benefits of MBSR therapy in reducing pain and improving sleep disorders in patients by mediating IL-β levels. Burrowes SAB *et al*. [35] reported that patients who underwent MBSR training demonstrated significant improvements in sleep quality, possibly related to reduced headache frequency. Cherkin *et al*. [36] reported that compared to usual care, MBSR therapy can reduce low back pain and may be regarded as an effective treatment for individuals suffering from chronic low back pain. MBSR therapy has shown promising results in improving sleep quality by reducing pain and decreasing the frequency of pain experienced by patients. Therefore, MBSR therapy can be considered a viable option for individuals with low back pain.

This study has several limitations that need to be addressed. Firstly, the small sample size and the selection of patients from our hospital might have introduced bias and limited the generalizability of the findings. Secondly, the short duration of the study prevented a comprehensive analysis of the long-term effect of MBSR therapy on pain management in patients with trauma and fractures. Finally, the impact of postoperative rehabilitation exercises was not assessed. In our future investigations, we plan to address these limitations by increasing the sample size and diversifying the sources of the study subjects.

## Conclusion

MBSR therapy can effectively alleviate ASD in patients with trauma and fractures. Additionally, this therapeutic approach can reduce the incidence of ASD and alleviate anxiety, depression, and negative emotions in patients. These positive effects collectively contribute to substantial improvements in sleep quality and overall well-being of patients.

## Availability of Data and Materials

Data to support the findings of this study are available on reasonable request from the corresponding author.
